# Myc-induced glutaminolysis bypasses HIF-driven glycolysis in hypoxic small cell lung carcinoma cells

**DOI:** 10.18632/oncotarget.16904

**Published:** 2017-04-06

**Authors:** Matilda Munksgaard Thorén, Marica Vaapil, Johan Staaf, Maria Planck, Martin E. Johansson, Sofie Mohlin, Sven Påhlman

**Affiliations:** ^1^ Translational Cancer Research, Department of Laboratory Medicine, Lund University, Medicon Village, Lund, Sweden; ^2^ Division of Oncology and Pathology, Department of Clinical Sciences Lund, Lund University, Medicon Village, SE Lund, Sweden; ^3^ Department of Oncology, Skåne University Hospital, SE Lund, Sweden; ^4^ Center for Molecular Pathology, Department of Laboratory Medicine, Lund University, Skåne University Hospital, Malmö, Sweden; ^5^ Current address: Biosciences Area, Division of Environmental Genomics and Systems Biology, Lawrence Berkeley National Laboratory, Berkeley, CA, USA

**Keywords:** small cell lung carcinoma (SCLC), hypoxia, hypoxia-inducible factor, MYC, glutamine

## Abstract

We previously demonstrated that small cell lung carcinoma (SCLC) cells lack HIF-2α protein expression, whereas HIF-1α in these cells is expressed at both acute and prolonged hypoxia. Here we show that low *HIF2A* expression correlates with high expression of *MYC* genes. Knockdown of *HIF1A* expression had no or limited effect on cell survival and growth *in vitro*. Unexpectedly, hypoxic ATP levels were not affected by HIF-1α knockdown and SCLC cell viability did not decrease upon glucose deprivation. In line with these *in vitro* data, xenograft tumor-take and growth were not significantly affected by repressed *HIF1A* expression. Glutamine withdrawal drastically decreased SCLC cell proliferation and increased cell death at normoxia and hypoxia in a HIF-independent fashion and the dependence on glutaminolysis was linked to amplification of either *MYC* or *MYCL*. Downregulation of *GLS* expression, regulating the first step of the glutaminolysis pathway, in *MYC/MYCL* overexpressing SCLC cells resulted in both impaired growth and increased cell death. Our results suggest that *MYC/MYCL* overexpression in SCLC cells overrides the need of HIF-1 activity in response to hypoxia by inducing glutaminolysis and lipogenesis. Targeting the glutaminolysis pathway might hence be a novel approach to selectively kill *MYC* amplified SCLC cells *in vivo*.

## INTRODUCTION

Small cell lung cancer (SCLC), representing about 15% of all lung cancers, frequently presents with advanced or disseminated stage of disease. The prognosis is poor and has changed little for the past decades. Novel therapeutic strategies against SCLC are urgently needed, thus providing a rationale for further studies of targetable tumorigenic mechanisms in this devastating disease. Herein, we aimed to clarify the importance of various metabolic pathways and the impact of hypoxia for SCLC cell proliferation and survival.

Tumors are generally poorly oxygenated due to high tumor cell proliferation rate and incomplete vascularization [1, 2]. The heterodimeric hypoxia-inducible transcription factor (HIF)-1 and HIF-2 primarily regulate the adaptive cellular response to hypoxia by inducing transcription of genes involved in e.g. cell survival, proliferation, angiogenesis and metabolism [3]. At low oxygen levels, HIF-α subunits become stabilized and dimerize with ARNT/HIF-1β and co-activators to form the transcription complexes that bind to hypoxia-response elements (HRE) in HIF target genes [3, 4]. We have previously found that hypoxic SCLC cells as well as tumor specimens lack expression of HIF-2α. In response to acute and prolonged hypoxia, only HIF-1α is stabilized in these cells. Despite the HIF-2 deficiency, SCLC cells seem to adapt properly at both modest and severe hypoxia as they survive and propagate under these conditions [5]. Interestingly, SCLC cells survive and proliferate at hypoxic conditions also when *HIF1A* expression is repressed [5], suggesting that SCLC cells can use alternative pathways for adaptation to hypoxic growth conditions *in vitro*. Here we show that tumor-take and tumor growth of xenografted SCLC cells in mice are not drastically impaired by knocking down *HIF1A* by shRNA.

As HIF-1 actively transcribes genes involved in metabolic pathways at hypoxia we have examined how HIF-1 reduction in SCLC cells affects metabolic gene transcription and tumor growth. A common feature of proliferating tumor cells is their high rate of glucose metabolism, which is further promoted by hypoxia. In non-transformed, differentiated cells, glucose is converted via glycolysis into pyruvate and further into acetyl-CoA that enters the tricarboxylic acid (TCA) cycle leading to efficient ATP production through oxidative phosphorylation. At oxygen shortage glycolysis is the major ATP source and pyruvate is primarily converted into lactate by HIF-driven lactate dehydrogenase A (LDHA) [6–9]. To maintain intracellular pH at hypoxic conditions the transporter protein SLC16A3/MCT4 pumps lactate out of the cell, also a HIF-driven process [10]. In addition to glucose, glutamine can be utilized to produce ATP, cellular building blocks as well as be involved in the synthesis of glutathione, regulating the redox status. In these pathways glutamine is primarily taken up by the transporter SLC1A5 and in the first step of glutaminolysis, glutamine is metabolized to glutamate by the enzyme glutaminase (GLS). Glutamate is then converted to α-ketoglutarate, which is further catabolized through the TCA cycle or undergo reductive carboxylation to produce citrate, a building block in lipid synthesis [7, 11–13]. During *de novo* lipogenesis the cytosolic citrate is cleaved by ATP citrate lyase (ACLY) into oxaloacetate and acetyl-CoA. The following steps in the fatty acid pathway are catalyzed by acetyl-CoA carboxylase alpha (ACACA) and fatty acid synthase (FASN), respectively [14]. Glutamine metabolism pathways have been described as important and potentially targetable in both lung cancer and other cancer types [15].

*MYC* overexpression stimulates glutamine metabolism through transcriptional activation of genes involved in glutaminolysis [16, 17], and high rate of glutaminolysis support cell viability and proliferation [18–20]. Interestingly, in tumors with *MYC* or *MYCN* overexpression, glycolysis is less important as compared to glutaminolysis in keeping the energy status and for survival, as has been reported in *MYCN* amplified neuroblastoma cells as well as in MYC-inducible B-cells [20, 21].

A subset of SCLCs carry a *MYC* gene (*MYC*, *MYCL, MYCN)* amplification [22–24], and we investigated the importance of glucose and glutamine for cell survival and proliferation in cells derived from such SCLCs. We found that *MYC* and *MYCL* amplified SCLC cells are dependent on glutamine, but not on glucose, and that long-lasting knockdown of HIF-1α in these cells does not affect cell growth and cell survival at hypoxic conditions. Our data suggest that *MYC* overexpression compensates for lack of HIF-1 activity in hypoxic SCLC and that targeting regulatory steps in the glutaminolysis and lipogenesis pathways might be novel strategies to eradicate *MYC* amplified tumor cells.

## RESULTS

### *MYC*/*MYCL* amplified SCLC lack HIF2A expression and proliferate and survive following *HIF1A* knockdown at hypoxia

**Figure 1:**
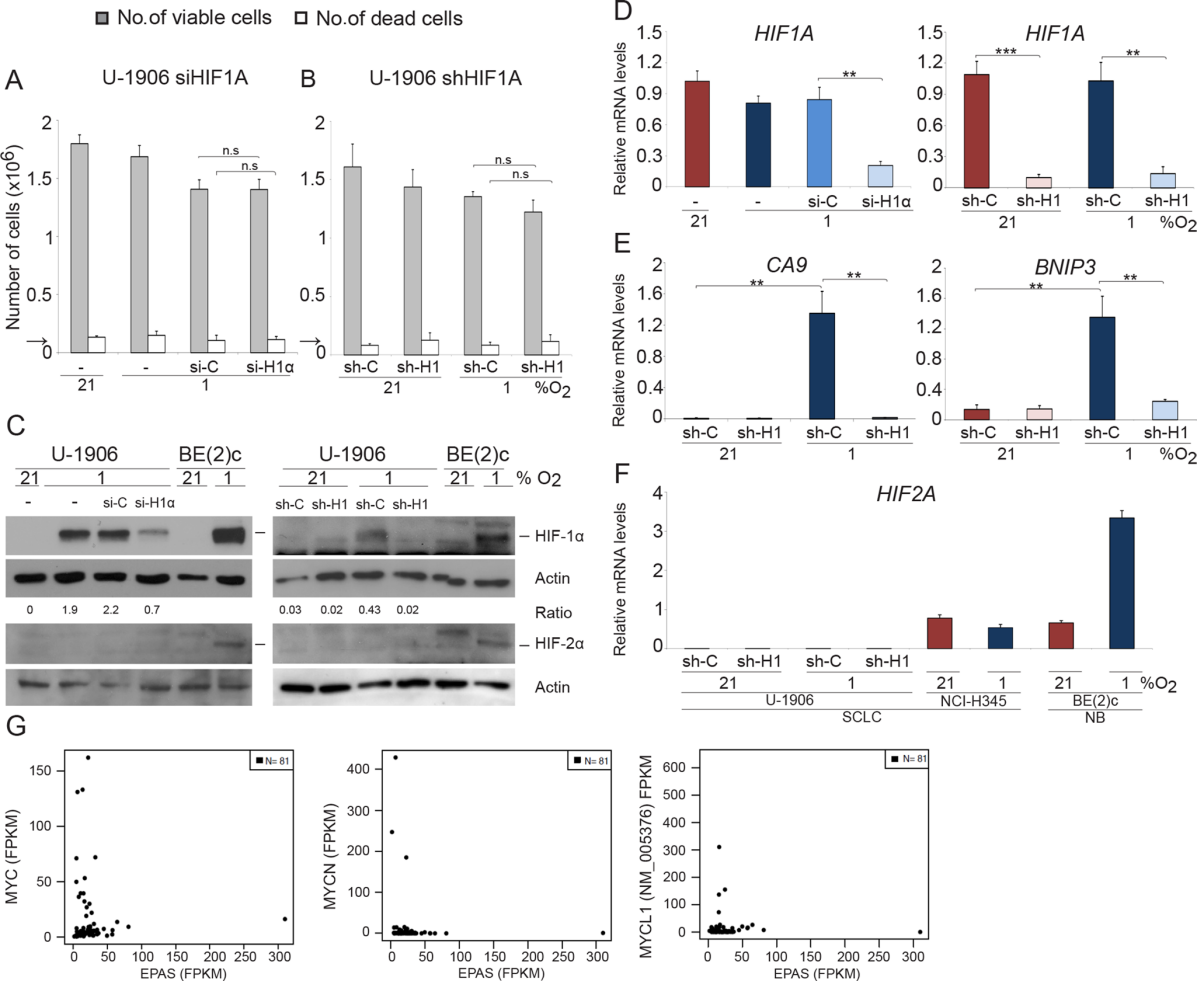
SCLC cells survive and proliferate at 1% oxygen despite *HIF1A* knockdown U-1906 cells were (**A**) transfected with siRNA (si-H1α) or lipofectamine alone (−) or (**B**) transduced with shRNA (sh-H1) against *HIF1A* or a non-targeting control (si-C and sh-C). The cells were cultured for 72 hours at 21% or 1% oxygen. The number of viable and dead cells was counted in triplicates. The arrows indicate number of cells seeded at day 0. (**C**) HIF-1α and HIF-2α western blot analyses of whole cell lysates. SK-N-BE(2)c neuroblastoma cells (BE(2)c) grown at 1% oxygen for 4 hours and 72 hours were used as positive HIF controls. Actin was used as loading control. The ratio between HIF-1α/Actin was calculated. (**D**–**F**) The relative mRNA expression levels of *HIF1A*, HIF2A, *BNIP3* and *CA9* were analyzed by qPCR and the expression data were normalized to three reference genes (*HPRT1, UBC, TBP*). Error bars show the standard deviation and 2-tailed unpaired Student's *t* test were performed. * indicates *p* < 0.05, ** < 0.01, *** < 0.001. (**G**) Expression of *MYC* (left panel), *MYCN* (center), and *MYCL1* versus *EPAS* for 81 SCLC tumors reported by George et al. (PMID:26168399).

The human SCLC cells U-1906 and U-1690 virtually lack *HIF2A* expression (Figure 1 and Supplementary Figure 1) suggesting a dependence on HIF-1 for adaptation and survival of these cells at hypoxia. However, knockdown of *HIF1A* using shRNA or siRNA had no or limited effects on U-1906 and U-1690 SCLC cell growth and survival at hypoxic (1% oxygen) growth conditions (Figure 1A, 1B and Supplementary Figure 2A, 2B). The two knockdown approaches robustly reduced *HIF1A* protein mRNA and HIF-1 activity as measured by impaired increase in expression of HIF target genes like *BNIP3* and *CA9* at hypoxia (Figure 1C–1E and Supplementary Figure 2C, 2D). We conclude that both approaches efficiently target *HIF1A* expression in SCLC cells and that reduction of HIF-1 activity did not impair growth of SCLC cells at hypoxic conditions. These results were not caused by an upregulation of *HIF2A* expression (Figure 1F).

SCLC cell lines frequently carry an amplified *MYC* gene and U-1906 and U-1690 cells are *MYC* and *MYCL* amplified, respectively (Supplementary Figure 1A, [25]). As *MYC/MYCL* amplified SCLC cells lack or have greatly reduced *HIF2A* expression [5] we investigated one of few available non-*MYC* amplified SCLC cell lines, NCI-H345, for both *MYC* and *HIF2A* expression. As shown in (Supplementary Figure 2 and 1F), expression of *MYC/MYCN/MYCL* in these cells was low and *HIF2A* expression was high when compared to *MYC*-amplified SCLC cells. This putative negative correlation between the expression of *MYC/MYCL/MYCN* and *HIF2A* is supported by data retrieved from the databank of a published expression analysis of 81 SCLC tumor specimens showing that tumors with high pan-MYC expression generally express low levels of *HIF2A* (Figure 1G; PMID: 26168399).

### Tumor growth is not significantly affected by reduced *HIF1A* expression in SCLC cell xenografts

**Figure 2:**
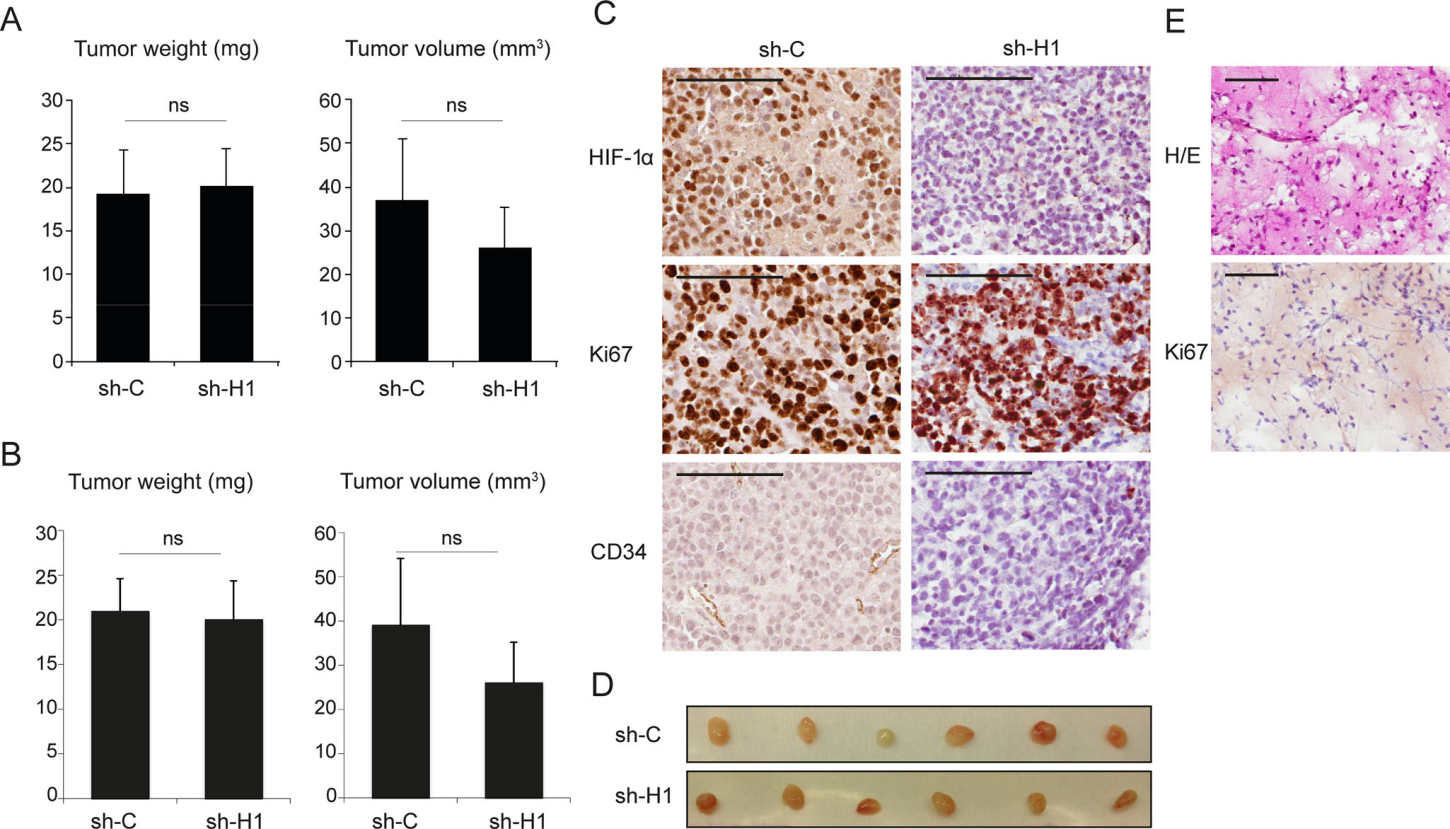
Repressed *HIF1A* expression does not significantly affect tumor growth in SCLC cell xenografts U-1906 cells, transduced with shRNA against *HIF1A* (sh-H1) or a non-targeting control (sh-C), were injected subcutaneously into nude mice. (**A**, **B**) Mean tumor specimen weight and tumor volume, 14 days after injection. A, all tumors (*n* = 12) and B, specimens with confirmed tumor growth (*n* = 11, see text and Panels in E). Data are presented as mean ± SEM and statistical significance was determined using Student´s *t*-test. Data is from one of two independent experiments (*n* = 6 for each group). (**C**) Sections of formalin-fixed paraffin-embedded xenografts were characterized immunohistochemically for expression of HIF-1α, Ki67 and CD34. (**D**) The corresponding dissected tumors are shown. (**E**) Example of a specimen, devoid of tumor cells, stained with hematoxylin-eosin (H/E) and anti-Ki67 antibody. Scale bars, 100 μm.

As SCLC cells have no or very low *HIF2A* expression we initially hypothesized that down-regulation of *HIF1A* expression by siRNA/shRNA treatment of SCLC cells *in vitro* would affect cell survival at hypoxic growth conditions, which we show is not the case [5]. Obviously, reduced *HIF1A* expression might not affect *in vitro* as severely as *in vivo* cellular growth and survival. To investigate *in vivo* effects of low overall HIF expression in SCLC cells we used transduced U-1906 cells as a nude mouse xenograft model based on the efficient knockdown of *HIF1A* expression and activity and minute HIF2A expression in these cells. Interestingly, palpable tumors were formed in all animals, irrespective of the *HIF1A* expression status (Figure 2 and Supplementary Figure 3). Histology combined with immunohistochemistry revealed however, that a few of the smallest lesions were apparently devoid of human tumor cells. Instead these lesions were composed of a loose connective tissue, derived from the host mice. Thin fibrous septa and a delicate capillary network were seen to invade into the former injection site. Scattered inflammatory cells, mainly lymphocytes, were also seen in the tissue. We interpret the histology as resulting from the initial organization of the matrigel by ingrowth of granulation tissue into the injection site attracted by matrigel components (Figure 2D and 2E). Taking also this observation into account, the tumor take was still not significantly affected by the *HIF1A* expression status. Out of total 17 injected animals in each group in three independent experiments, shC cells formed tumors in 14 while shHIF1A cells formed tumors in 12 animals. In addition, tumor size and weight did not differ significantly between shC and shHIF1A-transduced cells 14 or 27 days after injection (Figure 2A, 2B and Supplementary Figure 3A, 3B).

Immunohistochemical analysis revealed that tumors from both shC and shHIF1A transduced cells had high content of proliferating Ki67 positive cells (Figure 2C). In shC tumors, areas with strong HIF-1α positivity were frequent, while shHIF1A tumors contained few HIF-1α positive areas, and only with weak HIF-1α staining (Figure 2C). Furthermore, blood vessels as visualized by CD34 positivity were less abundant in shHIF1A tumors as compared to controls, suggesting that angiogenesis is impaired in these tumors due to low HIF activity. We conclude that SCLC cells have the ability to form tumors when HIF expression is substantially reduced (Figure 2).

### Hypoxia-induced glycolysis is HIF-1 dependent, but glucose deprivation does not affect SCLC cell survival at hypoxia

**Figure 3:**
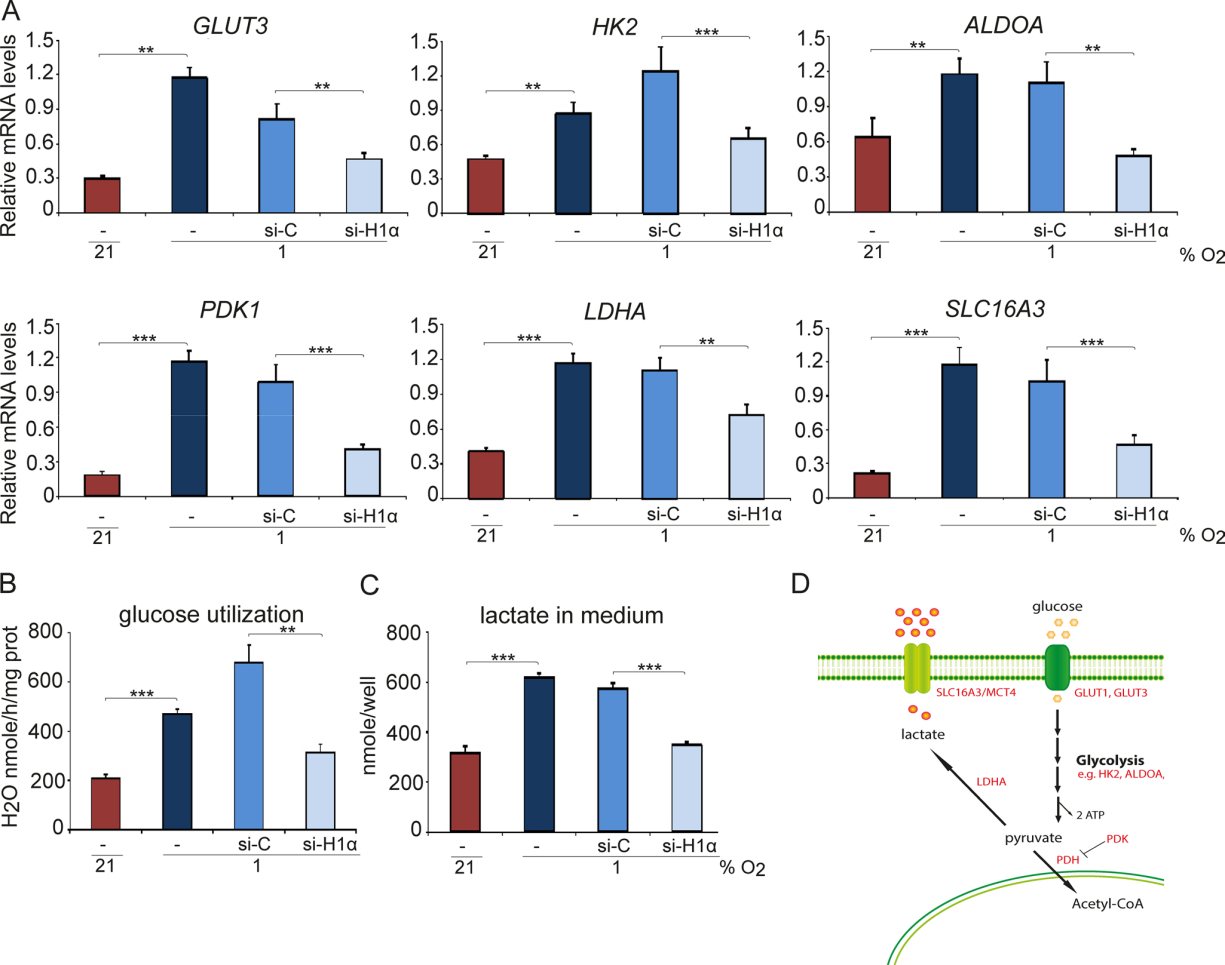
Hypoxia-induced glycolysis is HIF-1α dependent U-1906 cells were transfected with siRNA against *HIF1A* (si-H1α), a non-targeting siRNA as control (si-C) or lipofectamine alone (−). The cells were cultured at 21% or 1% oxygen for 72 hours. (**A**) The relative mRNA expression levels of *GLUT3, HK2, ALDOA, PDK1, LDHA* and *SLC16A3* were analyzed by qPCR and all expression data were normalized to three reference genes (*HPRT1, UBC, TBP*). (**B**) Glucose utilization in U-1906 cells and (**C**) lactate concentration in cell culture medium were measured. One representative experiment is shown; error bars show the standard deviation within triplicates. All statistical analyses were performed using 2-tailed unpaired Student's *t* test, * indicates *p* < 0.05, ** < 0.01, *** < 0.001. (**D**) Analyzed genes and their role in anaerobic glycolysis.

As the HIF deficient SCLC cells did survive *in vivo* and formed tumors, we next investigated the dependence on glucose metabolisms on cell division and survival as one major function of HIFs in the adaptive response to hypoxia is to shift the cellular energy production towards glycolysis and anaerobic metabolism. Hypoxia up-regulated the transcription of glycolysis genes in SCLC cells and knockdown of *HIF1A* by either siHIF1A or shHIF1A resulted in abrogation of hypoxia-induced expression of glycolysis-related genes, such as *GLUT3*, *GLUT1*, *HK2*, *ALDOA*, *PDK1*, *LDHA* and *SLC16A3* (Figure 3A and Supplementary Figure 4). In agreement, *HIF1A* knockdown decreased hypoxia-induced glucose utilization and lactate production (Figure 3B, 3C). However, expression of the analyzed genes was not reduced to the same levels as in normoxic control cells, possibly due to incomplete knock down of HIF-1 (Figure 1C). The results could also be explained by activation of non-HIF-driven pathways with capacity to regulate hypoxic glucose metabolism.

**Figure 4:**
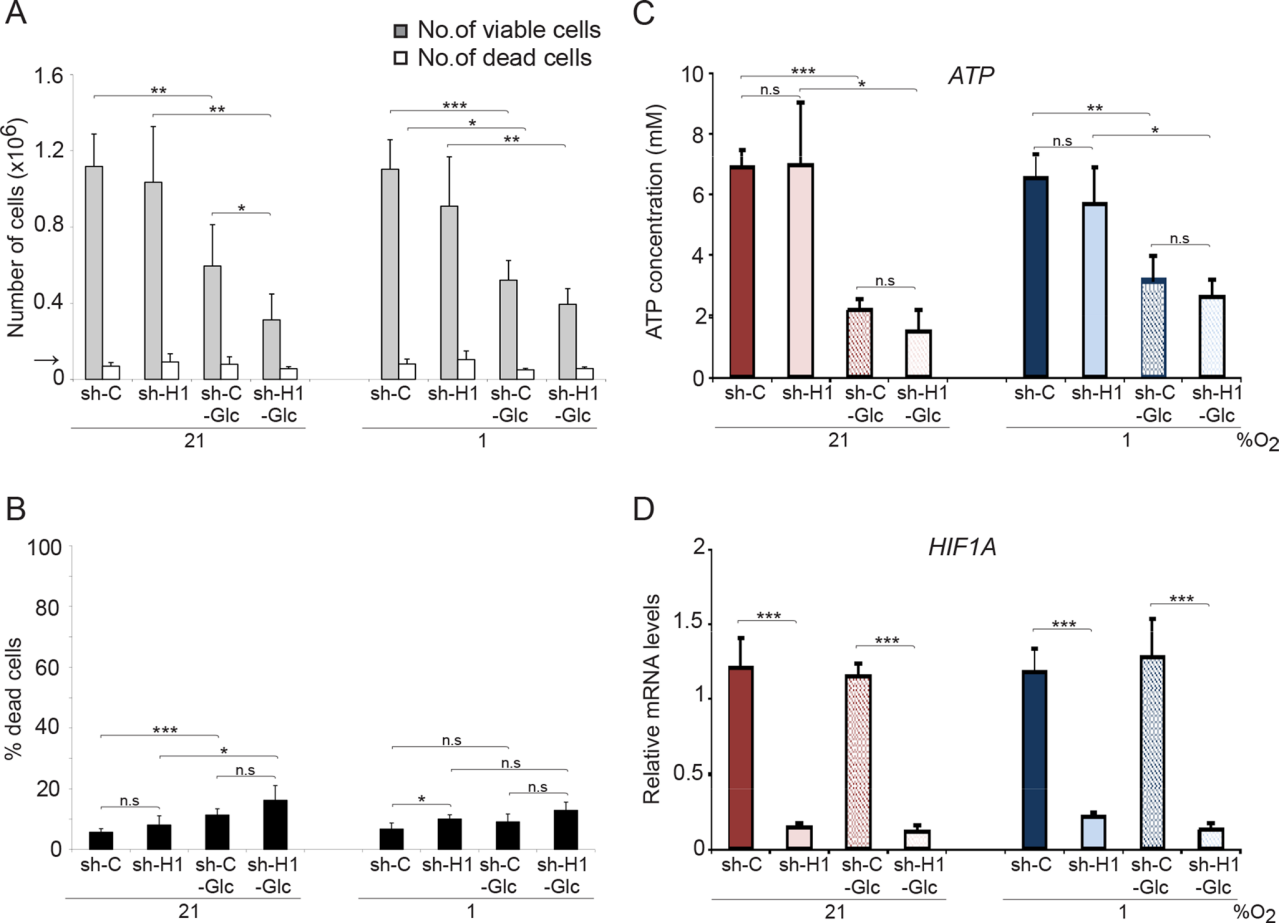
Glucose deprivation does not affect SCLC cell survival shHIF1A (sh-H1) and non-targeting shRNA (sh-C) transduced U-1906 cells were cultured in medium with (11,1 mM) or without glucose (−Glc) for 72 hours at 21% or 1% oxygen. (**A**) The number of viable and dead cells were determined and the arrow indicates the number of cells seeded at day 0. (**B**) The percentage of dead cells compared to total number of cells is also presented. (**C**) ATP levels were determined by a luciferin-luciferase based assay and (**D**) expression levels of *HIF1A* were analyzed by qPCR. Data are mean from three to five experiments; error bars show the standard deviation. All statistical analyses were performed using 2-tailed unpaired Student's *t* test, * indicates *p* < 0.05, ** < 0.01, *** < 0.001.

We next investigated the effect on cell division and survival after removal of glucose from the culture medium. Although proliferation was reduced by more than 50% at normoxia and hypoxia, glucose removal did not result in a substantially increased SCLC cell death (Figure 4A, 4B). Most importantly, there were no significant differences in growth rate or cell survival between shC and shHIF1A cells under hypoxic conditions despite efficient *HIF1A* knockdown (Figure 4D). Furthermore, glucose metabolism-related gene expression at hypoxic conditions decreased when *HIF1A* expression was repressed, but ATP levels did not differ significantly between shC and shHIF1A cells (Figures 3 and 4C). We conclude that the ATP levels in hypoxic SCLC cells are HIF-independent and that alternative mechanisms compensate for the decrease in glycolysis-generated ATP (Figure 3) when HIF-1 is repressed.

### SCLC cells are dependent on glutamine for growth and survival

**Figure 5:**
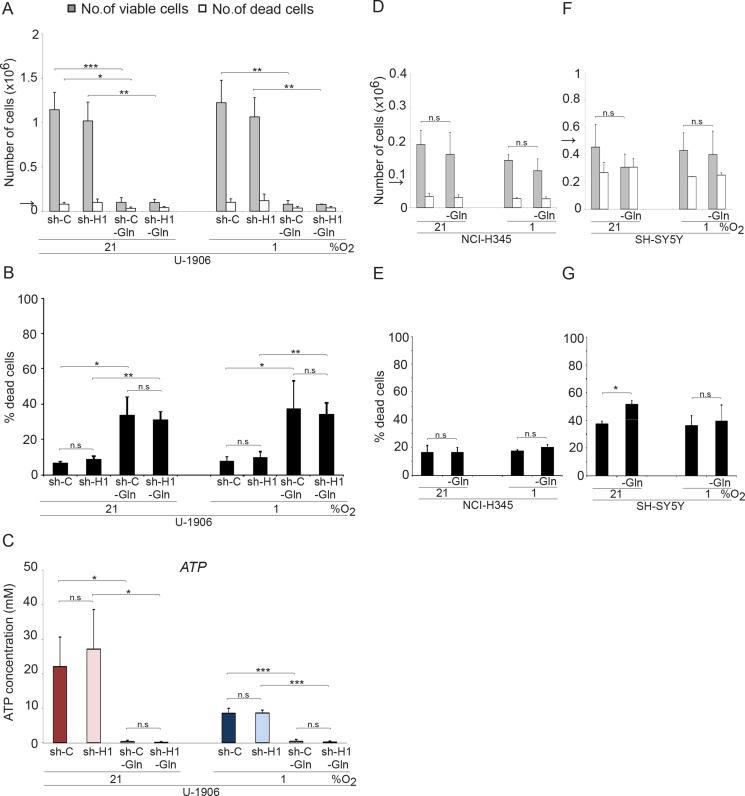
Glutamine withdrawal increases the number of dead cells in *MYC* amplified SCLC cells in a HIF-independent fashion The total number of viable and dead cells at 21% or 1% oxygen was counted after 72 hours. The arrows indicate the number of cells seeded at day 0. (**A**) The *MYC* amplified SCLC cell lines U-1906, transduced with shRNA (sh-H1) against *HIF1A* or a non-targeting control (sh-C), were grown in RPMI medium with (2 mM) or without glutamine (−Gln). (**D** and **F**) The non-*MYC* amplified NCI-H345 SCLC cells and SH-SY5Y neuroblastoma cells were cultured with or without glutamine. (**B**, **E**, and **G**) The percentage of dead cells compared to total number of cells is also presented. (**C**) ATP levels were determined by a luciferin-luciferase based assay. Data are mean from two to four experiments; error bars show the standard deviation. All statistical analyses were performed using 2-tailed unpaired Student's *t* test, * indicates *p* < 0.05, ** < 0.01, *** < 0.001.

*MYC* overexpressing tumor cells can utilize glutamine as energy source [20, 21]. In accordance, removal of glutamine from the medium of *MYC/MYCL* amplified SCLC cells efficiently blocked proliferation, increased cell death and the ATP pools were completely emptied both at normoxic and hypoxic conditions (Figure 5A–5C and Supplementary Figure 5). No significant differences in growth and survival between shC and shHIF1A cells were noted in the two cell lines tested. In contrast, glutamine deprivation did not affect growth or survival of non-*MYC* amplified NCI-H345 SCLC cells and non-*MYCN* amplified SH-SY5Y neuroblastoma cells (Figure 5D–5G) within the time frame tested. Taken into account published data from other tumor forms, our results suggest that *MYC* overexpression render also SCLC cells dependent on glutaminolysis for growth and survival at both normoxia and hypoxia.

**Figure 6:**
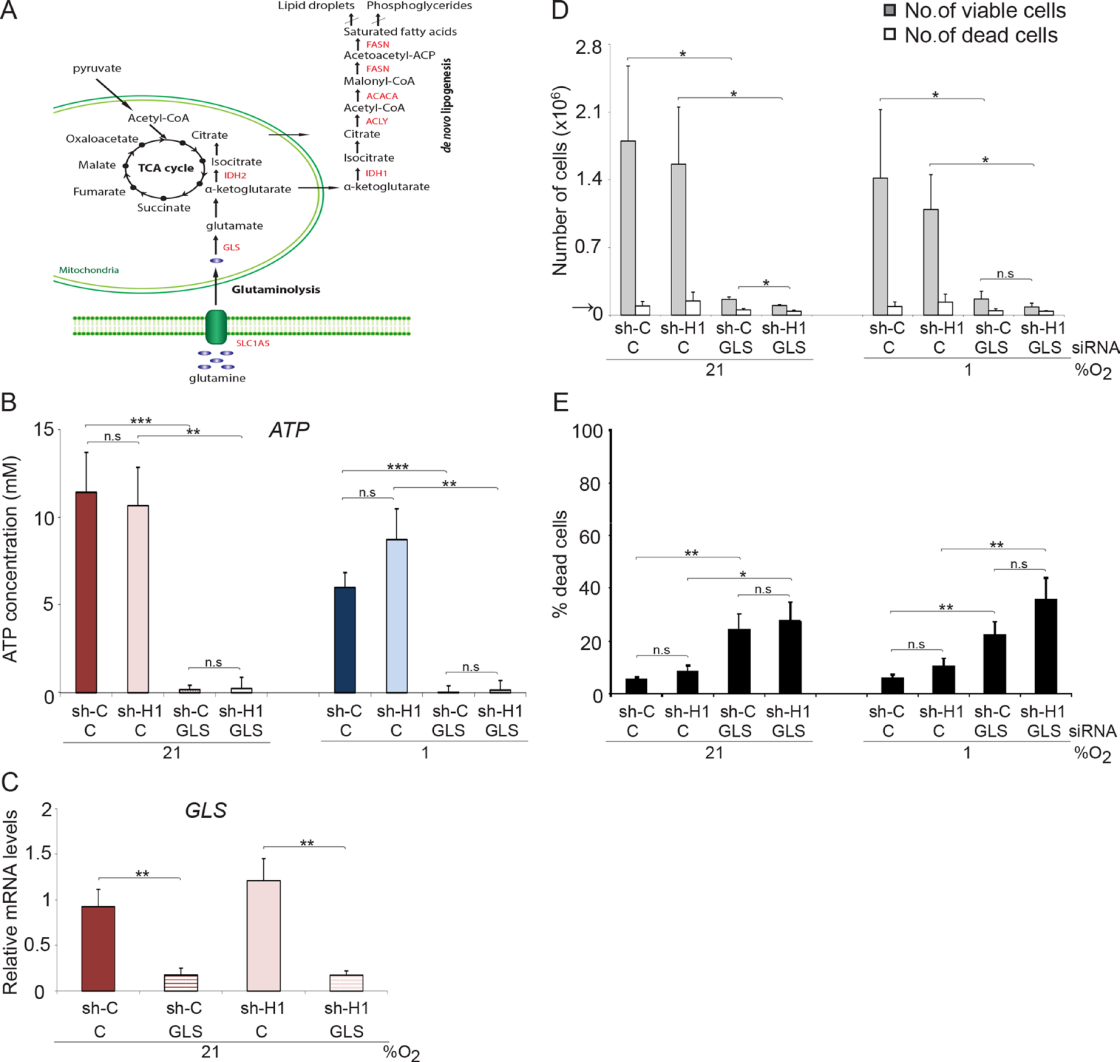
*GLS* knockdown affect proliferation and survival in SCLC cells (**A**) Schematic picture of intracellular glucose and glutamine metabolism. U-1906 cells transduced with shHIF-1α (sh-H1) or a non-targeting control (sh-C) cultured at 21% or 1% oxygen for 72 hours were transfected with siRNA against *GLS* or a non-targeting control. (**B**) ATP levels were determined by a luciferin-luciferase based assay. (**D**, **E**) The total numbers of viable and dead cells were counted and the percentage of dead cells compared to total number of cells is also shown. The arrows indicate the number of cells seeded at day 0. (**C**) U-1906 cells were transfected with siRNA against *GLS* or a non-targeting control for 24 hours at 21% oxygen and the relative mRNA expression levels of *GLS* was analyzed by qPCR. All expression data were normalized to three reference genes (*HPRT1, UBC, TBP*). Data are mean from three experiments; error bars show the standard deviation. All statistical analyses were performed using 2-tailed unpaired Student's *t* test, * indicates *p* < 0.05, ** < 0.01, *** < 0.001.

Our data imply that HIFs have a limited role in the control of survival and proliferation of *MYC* amplified SCLC cells. In search for a role of HIF-1 in these cells we next tested the influence of HIF-1 on the expression of genes involved in glutaminolysis. A slight increase in expression of *SLC1A5*, *GLS* and *IDH1* was noted in hypoxic SCLC cells when *HIF1A* was knocked down (Supplementary Figure 6A). In addition, the ammonia concentration increased slightly in *HIF1A* knocked down cells (Supplementary Figure 6C), further suggesting that HIF-1 has a somewhat negative impact on glutaminolysis at hypoxic conditions. Lipogenesis is linked to glutaminolysis (Figure 6A) and knocking down *HIF1A* results in a modest up-regulation of lipogenesis-related genes *ACLY*, *ACACA* and *FASN* at hypoxia (Supplementary Figure 6B). To directly test the importance of glutamine as an energy source and for maintaining *HIF1A* expression in *MYC/MYCL* amplified SCLC cells, we knocked down the expression of *GLS* (Figure 6C), the enzyme that converts glutamine to glutamate, in shC and shHIF1A SCLC cells (Figure 6A). Despite access to glucose and glutamine, *GLS* knockdown resulted in depletion of ATP pools and massive cell death (Figure 6B, 6D, 6E). Again, reduction of *HIF1A* expression had no significant effect on ATP levels or cell survival, suggesting that *MYC* overexpressing SCLC cells require glutamine, but not glucose, as energy source both at normoxia and hypoxia.

### *MYC* expression supports glutaminolysis and lipogenesis in *MYC* amplified SCLC cells

**Figure 7:**
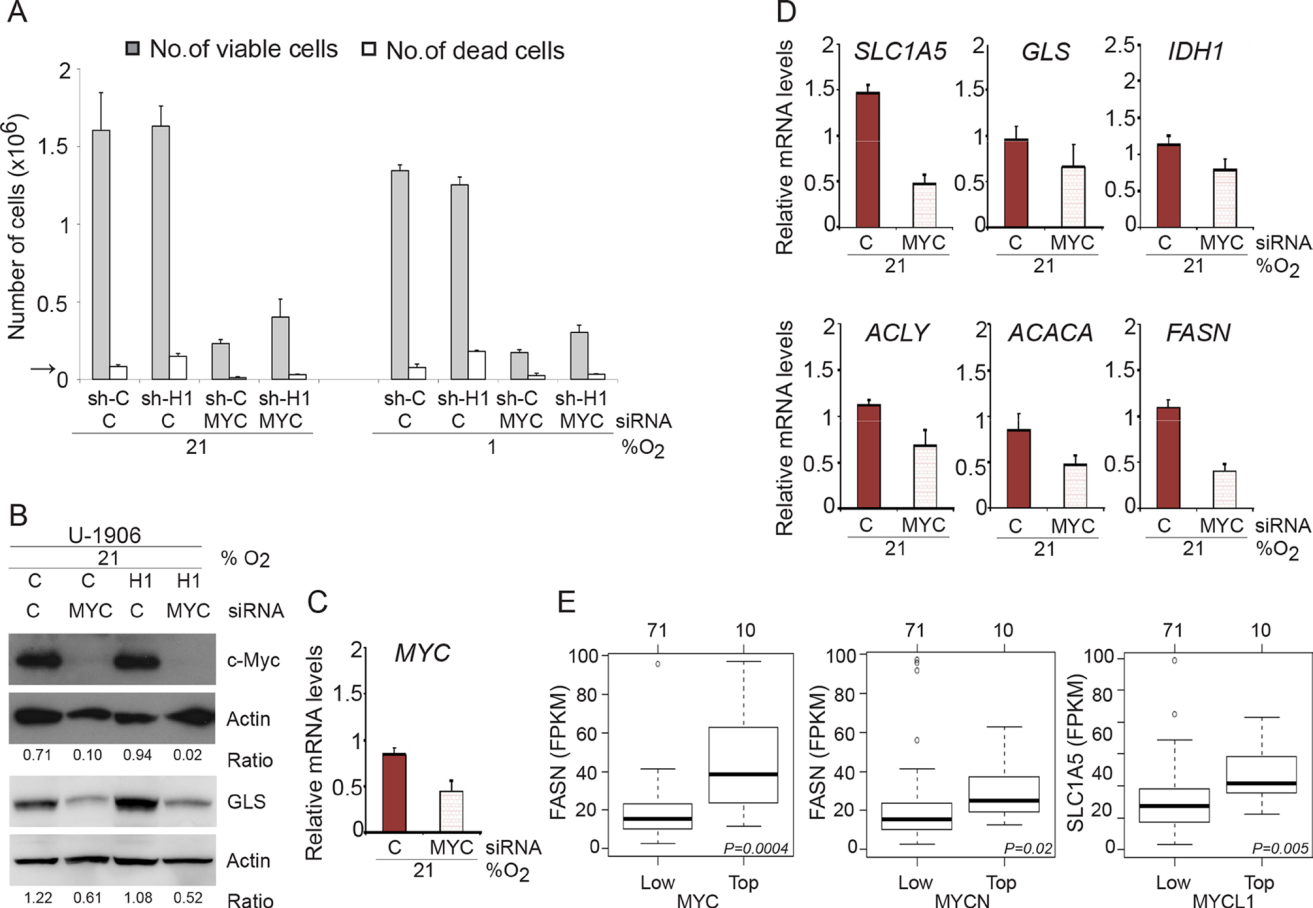
*MYC* knockdown affect proliferation in SCLC cells (**A**) U-1906 cells transduced with shHIF-1α (sh-H1) or a non-targeting control (sh-C) were transfected with siRNA against *MYC* and cultured for 72 hours at 21% or 1% oxygen. A non-targeting siRNA was used as control. The numbers of viable and dead cells were counted. The arrow indicates the number of cells seeded at day 0. (**B**) c-Myc and GLS western blot analyses of whole cell lysates from shHIF1A (H1) and non-targeting shRNA (C) transduced U-1906 cells. Actin was used as a loading control. The ratio c-MYC/Actin and GLS/Actin was calculated. (**C**, **D**) U-1906 cells were transfected for 24 hours at 21% oxygen. The relative mRNA expression levels of *MYC, SLC1A5, GLS, IDH1, ACLY, ACACA and FASN* were analyzed by qPCR and the expression data were normalized to three reference genes (*HPRT1, UBC, TBP*). One representative experiment is shown; error bars show the standard deviation within triplicates. (**E**) Expression of *FASN* for the 10 SCLC tumors with highest expression of *MYC* (left panel) or *MYCN* (centerpanel) versus 71 remaining SCLC tumors from George et al. (PMID:26168399). Right panel shows corresponding expression of *SLC1A5* for the 10 highest expressing *MYCL1* SCLC tumors versus remaining 71 tumors. *P*-values are calculated using Wilcoxon's test.

Myc plays an important role in driving proliferation of U-1906 SCLC cells, as demonstrated by the robust decrease in cell numbers after *MYC* knockdown (Figure 7A–7C). Proliferation was substantially decreased both at normoxia and hypoxia but cell death was not significantly affected by *MYC* knockdown. Interestingly, *MYC* knockdown resulted in a distinct reduction of GLS protein as well as a decrease in *Myc*-dependent expression of genes involved in glutaminolysis and lipogenesis (Figure 7B and 7D).

We next analyzed the mRNA expression of genes involved in glutaminolysis and lipogenesis in relation to *MYC/MYCL/MYCN* expression in the recently reported clinical SCLC databank (PMID: 26168399). These analyses revealed that high *MYC* mRNA expression correlates with high expression of *FASN*, the same result was seen for *MYCN* and *FASN*, while high *MYCL* expression primarily correlated to high *SLC1A5* expression (Figure 7E). Although the amplification of *MYC* genes in SCLC tumors are comparatively rare events (see e.g. PMID: 26168399) as compared to the frequent amplification of these genes in established cell lines, our data suggest that the phenotypes of cultured pan-*MYC* amplified SCLC cells mimics that of clinical SCLC and that *MYC* gene expression promotes glutaminolysis and lipogenesis.

## DISCUSSION

We have previously shown that *HIF2A* is not expressed in a clinical SCLC material and in cultured *MYC/MYCL*-amplified SCLC cells [5]. As the HIFs govern adaptation of cells to oxygen shortage we asked here how downregulation of HIF-1 in cultured SCLC cells would affect *in vivo* tumor initiation and tumor growth. We found unexpectedly that reducing HIF-1α protein levels in these cells had little or no effect on the tumor initiating capacity of xenografted SCLC cells. However, vascularization was impaired in the shHIF1A knocked down tumors suggesting that HIF-1 activity indeed was reduced in these *in vivo* growing cells. These results corroborate our *in vitro* data showing that the SCLC cells survived and proliferated at low and even extreme hypoxia (0.1% O_2_; [5]). Despite hypoxia-induced and HIF-1 dependent induction of genes regulating glycolysis in the examined SCLC cells, we could not detect HIF-1 driven effects on growth or survival at hypoxic conditions with or without glucose or glutamine in the culture medium. However, *MYC*/*MYCL* amplified SCLC cells, but not cells lacking *MYC* amplification, were dependent on the conversion of glutamine into glutamate by GLS for survival (Figure 6A), suggesting that high *MYC* gene expression overrides HIF-1 action at hypoxia. High *MYC* expression also promotes glutamine dependent lipogenesis as demonstrated in cultured cells and suggested by gene expression data in clinical SCLC specimens. In summary, HIF-1 seems not to be involved in controlling growth or survival in *MYC/MYCL* overexpressing SCLC cells at hypoxic conditions, and perhaps even more remarkable, HIF-1 seems not to be required for maintaining ATP levels in hypoxic SCLC cells with an amplified *MYC* gene.

As expected, *HIF1A* knockdown resulted in diminished expression of genes involved in hypoxia-induced glucose metabolism, as well as reduced glucose utilization and less transportation of lactate out of the cell. However, glycolysis was still inducible in the hypoxic shHIF1A cells and expression of glycolysis genes was only partially down-regulated, which in part could be explained by other factors, such as c-Myc, are stimulating this pathway also at hypoxic conditions [26–28]. Interestingly, the hypoxic ATP levels were not affected by *HIF1A* knockdown, despite reduced glucose metabolism. In line with a previous investigation showing that glucose deprivation was not affecting the viability of MYC-inducible B cells at hypoxia [20], our data suggests that hypoxic SCLC cells are not dependent on glucose for cell viability.

*MYC* and *MYCL* amplified SCLC cells were dependent on glutamine for proliferation and survival at both normoxic and hypoxic conditions, in a HIF-independent manner. Knockdown of *MYC* using siRNA decreased the proliferation substantially and knockdown of *GLS* resulted in both reduced propagation and increased cell death. In agreement with our results, previous studies have reported that enhanced c-Myc and N-Myc expression makes neuroblastoma and B-cells addicted to glutamine [17, 21] and suppression of glutamine metabolism, by inhibition of IDH1, IDH2 or GLS expression reduces cellular proliferation and tumor growth [20, 29–31]. Gao et al have demonstrated that c-Myc transcriptionally repress miR-23, which leads to an enhanced expression of the target protein GLS [16]. In line with this, we have shown that c-Myc knockdown leads to a modest decrease in GLS mRNA levels whereas a distinct reduction of the GLS protein level in the SCLC cells was shown.

Both HIF and Myc are involved in tumor metabolism and interactions have been reported, but this crosstalk seems to be very complex. Our results indicate that Myc overexpression overrides the need for HIF-1 for cell survival and propagation in response to hypoxia by inducing glutamine metabolism and *de novo* lipogenesis. We have shown that *MYC* amplified SCLC cells are not expressing HIF-2, and the most straightforward assumption would be that these cells become highly dependent on HIF-1 for their survival at hypoxia. This appears not to be the case, although we technically have not been able to fully knockdown *HIF1A* expression, leaving minute residual HIF-1a levels that theoretically could be enough to maintain and activate survival pathways. The alternative explanation, that additional pathways are activated by hypoxia working in parallel with the HIFs, cannot be ruled out here but needs to be experimentally proven. One approach to achieve a cleaner system to study would be to remove *HIF1A* (and *HIF2A*) by CRISPR technology, although we do not expect dramatic differences in outcome compared to the result presented here. Another approach would be to unravel HIF-independent mechanisms that protect the hypoxic, HIF-1α knocked down, SCLC cells. Clearly, there are more ancestral mechanisms protecting cells at oxygen shortages, like the unfolded protein response mechanism, which we previously showed becomes activated in the SCLC cells when culturing them at 0.1% oxygen [5]. Other mechanisms to investigate include ancestral modes of energy production in the absence of oxygen where fumarate respiration is a key step [32].

In summary, HIF-1 seems not to be involved in controlling growth or survival of hypoxic *MYC/MYCL* overexpressing SCLC cells and reduction of HIF-1 activity does not deplete ATP pools at hypoxia. Instead, sufficient amount of ATP for cell survival is produced via glutaminolysis in these cells and they are therefore dependent on glutamine for cell viability and propagation at normoxic and hypoxic conditions. The conversion of glutamine to glutamate by GLS is crucial for the survival of *MYC/MYCL* overexpressing SCLC cells and we propose that the glutaminolysis pathway is a potential target for this subgroup of SCLCs.

## MATERIALS AND METHODS

### Cell culture

The *MYC*/*MYCL* amplified human SCLC cell lines U-1690, U-1906, U-2020 and U-1285 were cultured in RPMI-1640 medium (Thermo Scientific HyClone, South Logan, UT) supplemented 10% FCS (EuroClone Ltd., UK), 100 units/ml penicillin and 100 μg /ml streptomycin (PEST) (Gibco, Paisly, UK). The cell lines were kind gifts from Dr. Kenneth Nilsson. These cell lines were established and characterized by Drs. Jonas Bergh and Kenneth Nilsson, Dept Pathology, Uppsala University [25]. The non-*MYC* amplified [33] SCLC cells NCI-H345 (ATCC, Manassas, VA) were grown in serum-free HITES medium; Dulbecco´s medium: Ham´s F12 (50:50) (Gibco) supplemented with insulin (0,005 mg/ml), transferrin (0,01 mg/ml), sodium selenite (30 nM), hydrocortisone (10 nM), β-estradiol (10 nM), HEPES (10 mM) and L-glutamine (2 mM). The human neuroblastoma cell lines SK-N-BE(2)c and SH-SY5Y were cultured in minimal essential medium (Thermo Scientific HyClone) supplemented with 10% FCS (EuroClone Ltd), 100 units/ml penicillin and 100 μg/ml streptomycin (PEST) (Gibco). The cell lines were kind gifts from Drs. June Biedler, Memorial Sloan Kettering Cancer Center and Robert Ross, Fordham University, Bronx, NY.

As part of our laboratory routines, all cell lines were regularly replaced on a tri-monthly basis, and screened for presence of mycoplasma infections. Critical gene expression patterns (e.g. for SCLC cells, *MYC, MYCL*, and lack of *HIF2A*, for neuroblastoma cells *MYCN*, *TH*, *CHGA*) and morphology were continuously monitored. In indicated experiments, cells were grown in medium without glucose (Gibco) or without glutamine (Gibco). For hypoxia, cells were cultured at 1% oxygen in a humidified oxygen-regulated chamber, H35 Hypoxystation (Don Whitley Scientific, Shipley, UK).

### Animal procedure and immunohistochemistry

Female athymic mice (NMRI-Nu/Nu strain; Taconic, Lille Skensved, Denmark) were housed in a controlled environment and all procedures were carried out in accordance with the regional ethical committee for animal research (Approval No. M331-11). U-1906 cells (5 × 10^6^) were prepared in 100 μl Matrigel:PBS (2,3:1). Mice were anesthetized by isoflurane inhalation and cells were injected subcutaneously on the right flank. The mice were sacrificed 2 or 4 weeks after injection, tumors were measured (V = (pi*l*(s)^2)/6 mm^3^; l = long side, s = short side) and weighed, in three independent experiments, before being snap-frozen for further analyses. Sections of formalin-fixed, paraffin embedded U-1906 xenograft tumors were, after antigen retrieval using PT Link (DAKO A/S, Glostrup, Denmark), stained for anti-CD34 (1:800, rat monoclonal, Santa Cruz Biotechnology, Santa Cruz, CA), anti-HIF-1α (1:50, mouse monoclonal, BD Biosciences, San José, CA) and anti-Ki67 (1:200, mouse monoclonal, DAKO A/S) using DAKO Autostainer Plus (DAKO A/S).

### Lentiviral transduction and siRNA transfections

For stable knockdown of *HIF1A* in SCLC cells, lentiviral shHIF1A particles carrying turboGFP and puromycin-resistance were used. U-1690 and U-1906 cells were seeded in a concentration of 50,000 cells/ml and 30,000 cells/ml respectively in 2 ml RPMI medium supplemented with 10% FCS. After 48 hours, the medium was replaced with polybrene (3 μl/ml) containing medium. Lentiviral shRNA were added to the cells to obtain a MOI of 2.5. Three different shRNA targeting *HIF1A* (SMART vector 2.0, Thermo Scientific, VSH5417, clones SH-004018-01, SH-004018-02, SH-004018-03) and a non-targeting control (SMART vector 2.0, Thermo Scientific, VSC5417) were used. 24 hours after transduction the cells were reseeded in selection medium containing puromycin (2 μg/ml).

For transient knockdown, U-1906 and U-1690 cells were seeded approximately 48 hours before transfection, in PEST free medium. The transfections were carried out in Opti-MEM (Gibco) with Lipofectamine 2000 as transfection reagent (Invitrogen/Life Technologies, Carlsbad, CA) for 7 hours, before medium was changed. Adherent cells were treated with Lipofectamine 2000 reagent only or transfected with the following siRNA; HIF1A (Applied Biosystems), MYC (Applied Biosystems), GLS (Applied Biosystems) or a non-targeting siRNA (Applied Biosystems) at a concentration of 50 nM. To improve knockdown efficiency the transfection procedure was repeated the following day. The cells were reseeded 1 day after the transfections and cultured for additional 72 hours at 21% or 1% oxygen before analyses.

### Quantitative real-time PCR

For quantitative real-time PCR (qPCR) experiments, RNA extraction was performed with RNeasy kit (Qiagen, Hilden, Germany) or isolated automatically using NorDiag Arrow kits (NorDiag, Dublin Ireland). cDNA synthesis and qPCR reactions with SYBR Green PCR master mix (Applied Biosystems, Warrington, UK) were performed as previously described [34]. The comparative Ct method to quantify relative mRNA was used [35]. For normalization of expression levels, three reference genes (*HPRT1*, *TBP* and *UBC*) were used, selected based on geNorm. Each reaction was performed in triplicate and primers were designed using Primer Express (Applied Biosystems, Foster City, CA) or Primer3; sequences are listed in (Supplementary Table 1).

### Western blot analyses

Western blot analyses with whole-cell RIPA buffer protein lysates were separated by SDS-PAGE and blotted onto Hybond-C-Extra nitrocellulose membranes (Amersham Bioscience, Little Chalfont, UK) or onto Trans-Blot Turbo Mini PVDF membranes (BioRad). Following primary antibodies were used after blocking: anti-HIF-1α (1:500, rabbit polyclonal, Millipore, CA) anti-HIF-2α (1:500, rabbit polyclonal Abcam), anti-c-Myc (1:100, mouse monoclonal Santa Cruz), anti-GLS (1:500, rabbit polyclonal, Proteintech), anti-SDHA (1:2,000, mouse monoclonal Abcam) and anti-β-Actin (1:500, mouse monoclonal Santa Cruz). The proteins were detected by using HRP-conjugated secondary antibodies; anti-mouse IgG (1:5,000, GE healthcare, Buckinghamshire, UK) and anti-rabbit IgG (1:5,000, GE healthcare) and EZ-ECL chemiluminescence detection kit (Biological Industries, Israel).

### Cell viability assay

For cell viability assay, the automated cell-counting system NucleoCounter NC 3000 (ChemoMetec, Allerød, Denmark) was used according to the manufactures protocol. To determine the number viable and dead cell, solution 13 containing Acridine Orange and DAPI was used. Acridine Orange stains all cells in the cell suspension whereas DAPI stains the non-viable cells. The experiments were performed in triplicates.

### Glucose utilization, lactate, ATP and ammonia assay

Cells were cultured for 72 hours at normoxic or hypoxic conditions. Cell culture medium was collected and used for lactate measurements using a colometric lactate assay kit (Biovision, Mountain View, CA), according to the manufactures recommendation. Cell culture plates were washed once with PBS and assay buffer containing 11.1 mM glucose (D-glucose+3H-5-D-glucose tracer) were added to each well and incubated for 1 hour at 37°C. The reaction was stopped by adding 60% trichloroacetic acid (TCA) to the wells. Blanks were prepared by adding 60% TCA to the wells prior addition of assay buffer. The cells were then scraped and 500 μl of lysates were transferred to Eppendorf tubes, which were placed inside scintillation vials containing 500 μl H_2_O, and incubated at 56°C overnight. The vials were then cooled down for 30 min. The Eppendorf tubes were removed and the scintillation liquid was measured in a β-counter.

For ATP measurement, the cells were harvested and sonicated for 20 seconds in lysis buffer containing 200 mM NaCl, 2 mM EDTA, 50 mM Tris and 1% Triton-X100. ATP was measured by a luciferin-luciferase based assay (BioThema, Sweden), following the manufacturer´s instructions. Medium from cells cultured at 21% or 1% oxygen were collected and ammonia concentrations were measured by Ammonia Assay Kit (Sigma Aldrich, St. Louis, MO).

### Gene expression analysis of SCLC tumors

RNAseq data for 81 SCLC tumors were obtained from (PMID: 26168399) as fragments per kilobase of exon per million fragments mapped (FPKM) expression values.

### Statistical analyses

*In vitro* data are reported as mean +/− SD and *in vivo* data are showed as mean +/− SEM. Group comparisons were made using a 2-tailed unpaired Student's *t*-test and statistical significance was set at levels of *p* < 0.05, *p* < 0.01 and *p* < 0.001.

## SUPPLEMENTARY MATERIALS FIGURES AND TABLE


