# Supplementary material for: Myc-induced glutaminolysis bypasses HIF-driven glycolysis in hypoxic small cell lung carcinoma cells
Source: Oncotarget. 2017 Apr 6;8(30):48983–95. doi: 10.18632/oncotarget.16904 (PMC5564742; doi:10.18632/oncotarget.16904)
Supplement: Supplementary file 1 [file oncotarget-08-48983-s001.pdf]

Myc-induced glutaminolysis bypasses HIF-driven glycolysis in hypoxic small cell lung carcinoma cells

Supplementary Materials

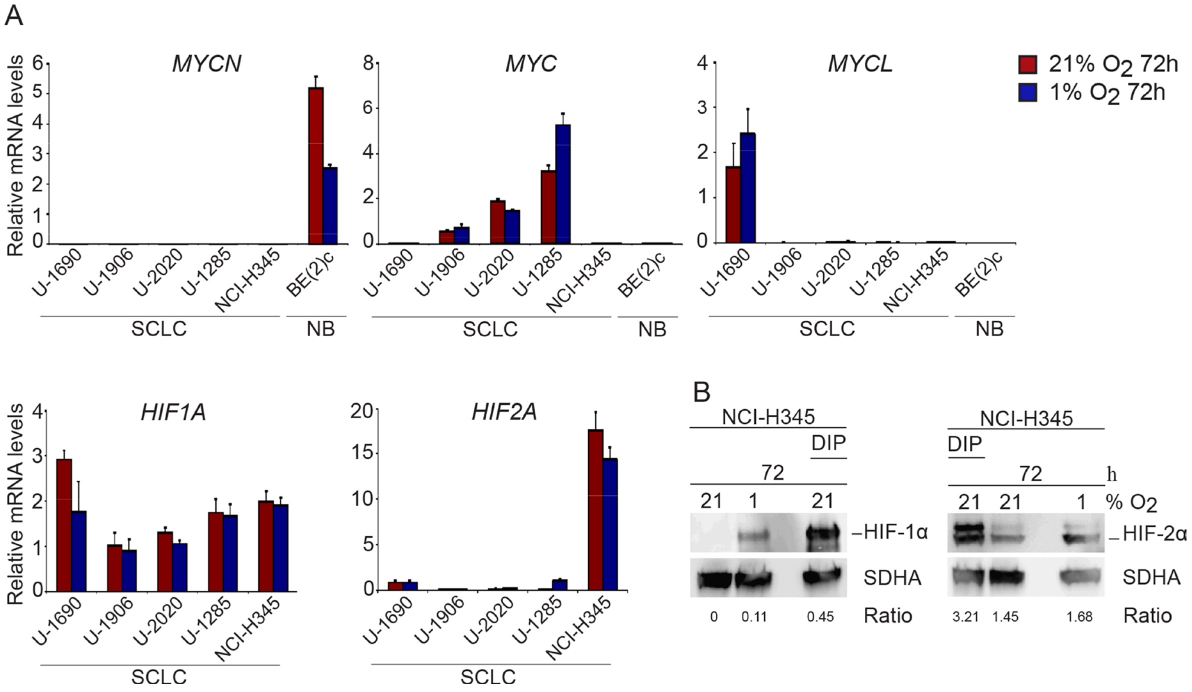

Supplementary Figure 1: *MYCN, MYC, MYCL, HIF1A* and *HIF2A* expression in SCLC cell lines. (**A**) The SCLC cells lines U-1690, U-1906, U-2020, U-1285 and NCI-H345 and the neuroblastoma cell line SK-N-BE(2)c were cultured at 21% or 1% oxygen for 72 hours. The mRNA levels were normalized to three reference genes (*HPRT1, UBC, TBP*) and data is one representative experiment; error bars show the standard deviation within triplicates. (**B**) HIF-1α and HIF-2α western blot analyses of whole cell lysates. DIP treated cells grown at 21% oxygen for 4 hours were used as a positive control. SDHA was used as loading control and ratio between HIF-1α/SDHA and HIF-2α /SDHA was calculated.

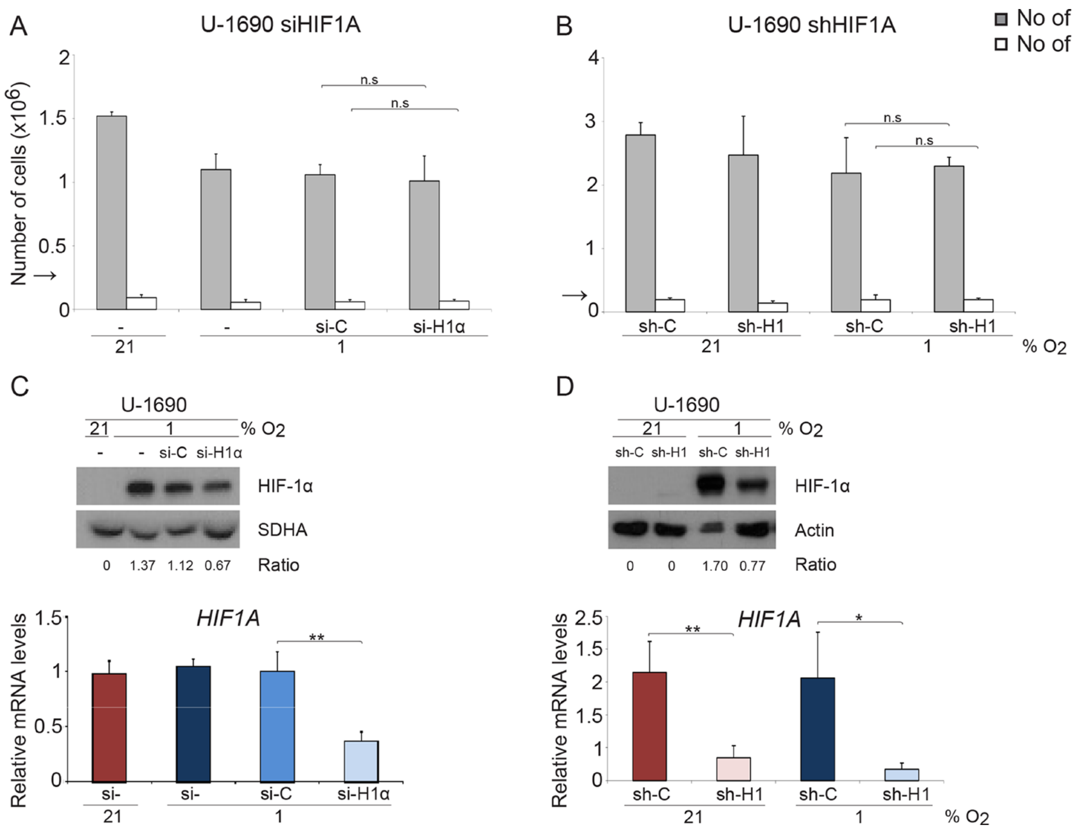

Supplementary Figure 2: SCLC cells survive well at 1% oxygen despite *HIF1A* repression. (**A**) U-1690 cells were transfected with siRNA against *HIF1A* (si-H1α) or a non-targeting siRNA (si-C) or treated with lipofectamine alone (−). (**B**) U-1690 cells were transduced with shRNA against *HIF1A* (sh-H1) or a non-targeting control (sh-C). The cells were cultured at 21% or 1% oxygen for 72 hours. The number of viable and dead cells was counted and the arrow indicates the number of cells seeded at day 0. (**C**, **D**) HIF-1α protein levels were analyzed by immunoblotting and SDHA were used as loading control. Ratio between HIF-1α and SDHA was calculated. mRNA expression levels of *HIF1A* were analyzed by qPCR and expression data were normalized to three reference genes (*HPRT1, UBC, TBP*). Error bars show the standard deviation and all statistical analyses were performed using 2-tailed unpaired Student's *t* test, * indicates $p < 0.05$, ** < 0.01, *** < 0.001.

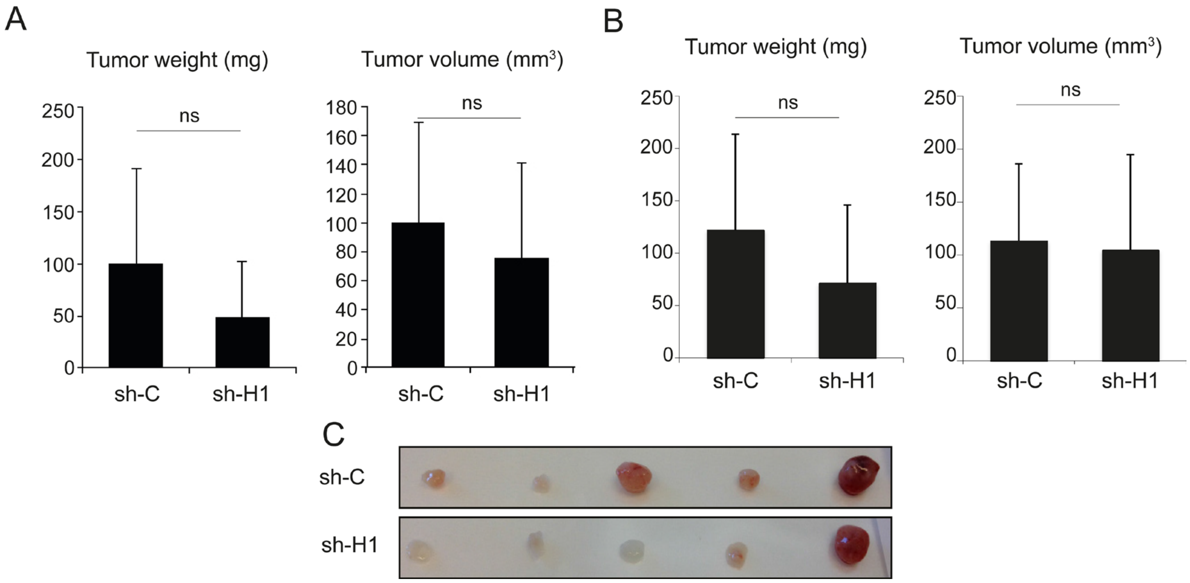

Supplementary Figure 3: Tumor size and tumor volume is not significantly affected by *HIF1A* repression in SCLC cell xenografts. The human U-1906 SCLC cells transduced with shRNA against *HIF1A* (sh-H1) or a non-targeting control (sh-C) were xenotransplanted into nude mice. 27 days after injection (n = 5 for each group), mean tumor specimen weight and tumor volume were measured in (**A**) all palpable tumors and (**B**) in specimens with confirmed tumor growth. Statistical analyses were performed by using Student´s *t*-test and error bars indicate SEM. (**C**) Representative pictures of xenograft tumors are shown.

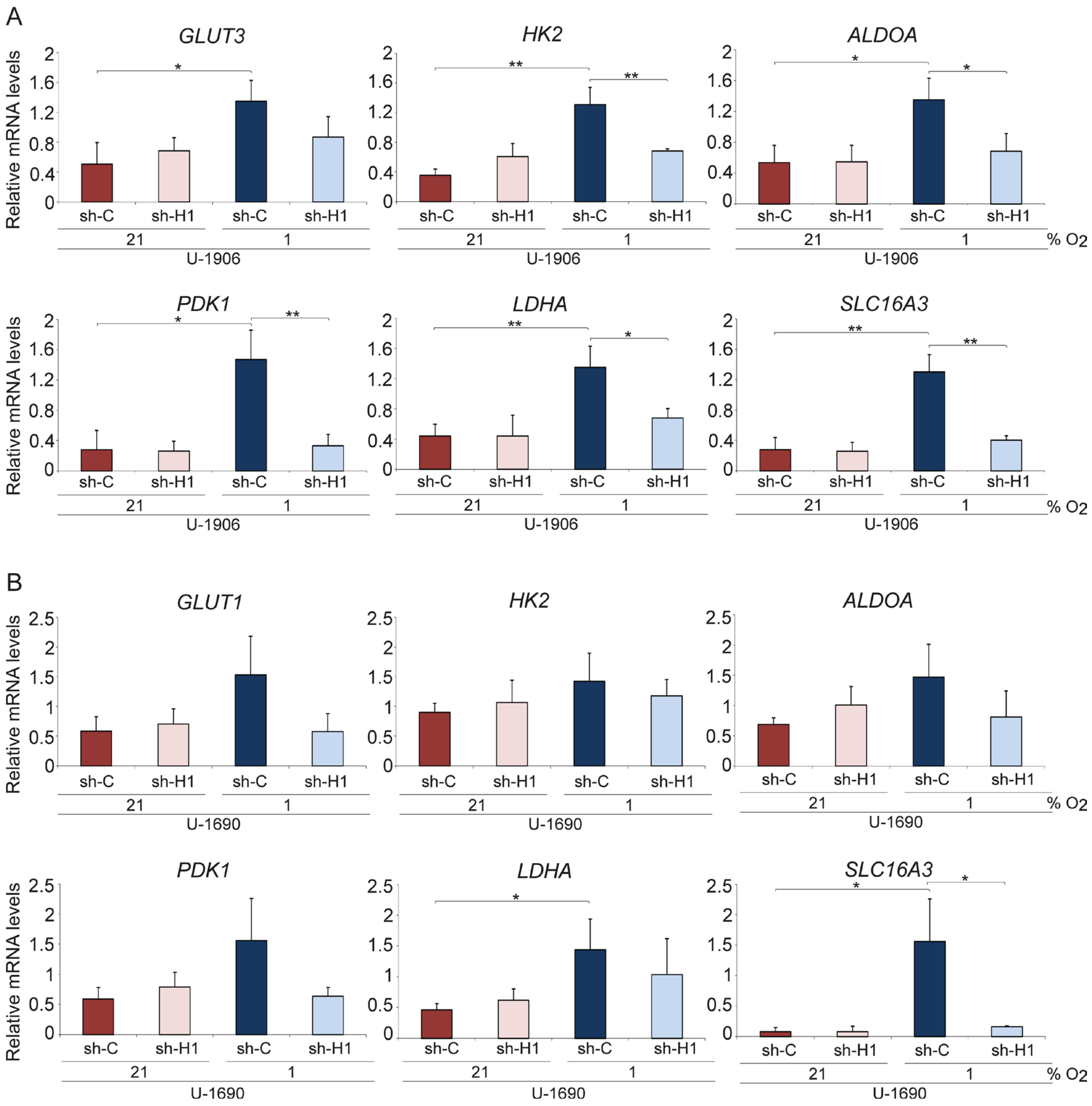

Supplementary Figure 4: In *HIF1A* repressed SCLC cells the anaerobic glycolysis is diminished. The SCLC cells, (**A**) U-1906 and (**B**) U-1690, transduced with shRNA against *HIF1A* (sh-H1) or a non-targeting control (sh-C) were cultured at 21% or 1% oxygen for 72 hours. The relative mRNA expression levels of indicated glycolytic genes were analyzed by qPCR and all expression data were normalized to three reference genes (*HPRT1*, *UBC*, *TBP)*. Data are mean from three experiments; error bars show the standard deviation. All statistical analyses were performed using 2-tailed unpaired Student's *t* test, * indicates $p < 0.05$, ** < 0.01, *** < 0.001.

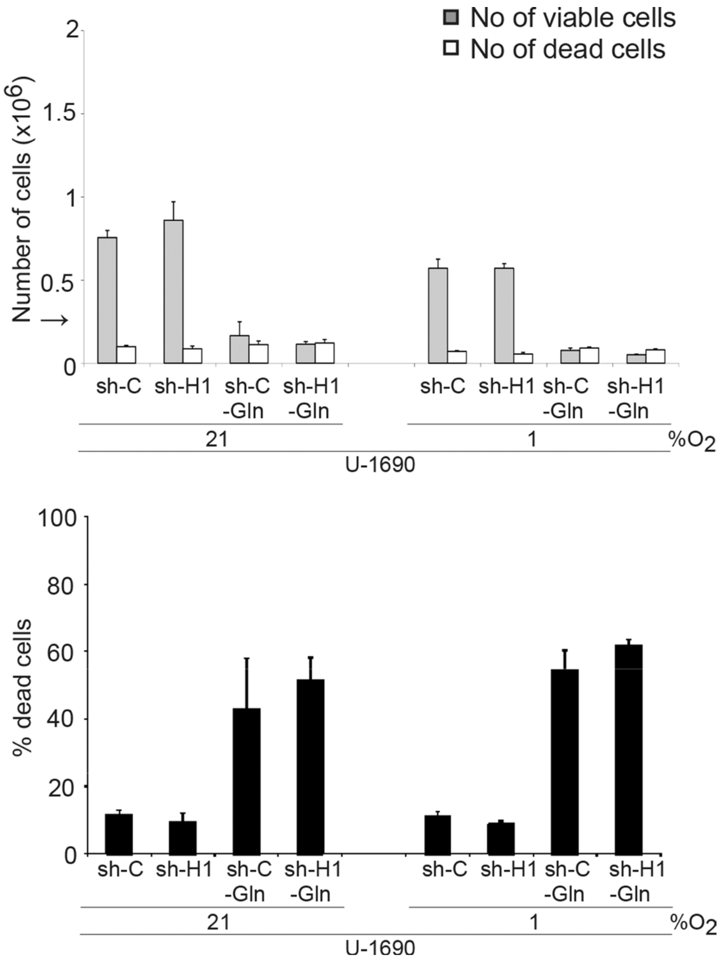

Supplementary Figure 5: Glutamine withdrawal increases the number of dead cells in *MYCL* amplified SCLC cells. The *MYCL* amplified U-1690 SCLC cells transduced with shRNA (sh-H1) against *HIF1A* or a non-targeting control (sh-C), were grown in RPMI medium with (2 mM) or without glutamine (-Gln). The total numbers of viable and dead U-1690 cells were counted after 72 hours culturing at 21% or 1% oxygen. The arrows indicate the number of cells seeded at day 0. The percentage of dead cells compared to total number of cells is also presented. Data is one representative experiment; error bars show the standard deviation within triplicates.

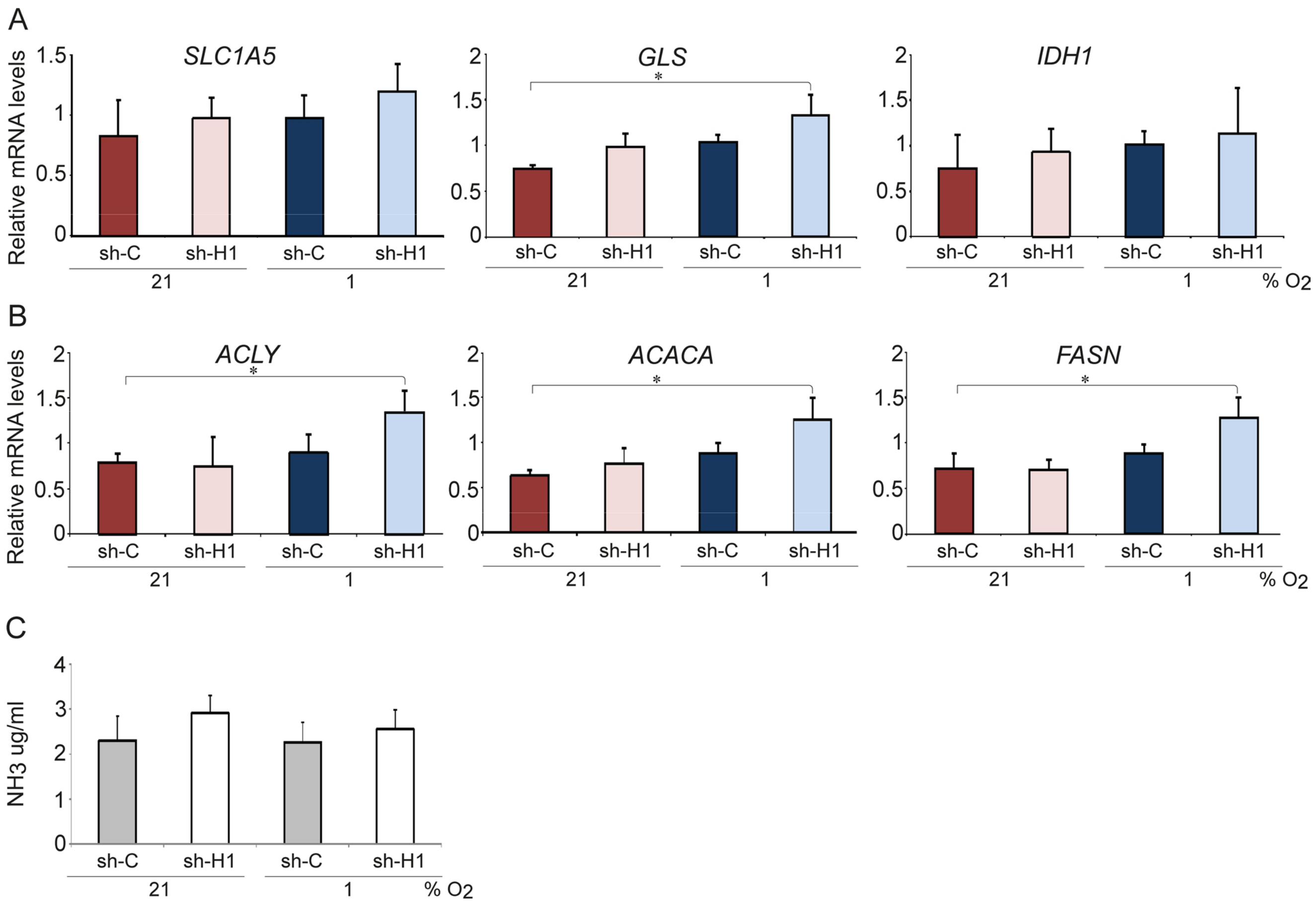

Supplementary Figure 6: Induced expression of genes involved in glutaminolysis and lipogenesis in *HIF-1A* repressed SCLC cells. U-1906 cells transduced with shHIF1A (sh-H1) or a non-targeting control (sh-C) were cultured for 72 hours at 21% or 1% oxygen. (**A** and **B**) The relative mRNA expression levels of *SLC1A5, GLS, IDH1, ACLY, ACACA* and *FASN* were analyzed by qPCR. All expression data were normalized to three reference genes (*HPRT1, UBC, TBP*). Data are mean from three experiments. Error bars show the standard deviation and 2-tailed unpaired Student's *t* test were performed, * indicates $p < 0.05$. (**C**) Secreted NH_3 levels in cell culture medium was spectrophotometrically measured. Data are mean of two experiments; error bars show the standard deviation within the two experiments.

Supplementary Table 1: Nucleotide sequences of primers used for Q-PCR reactions

Primers	Primer sequence
ACACA	f: 5′-cttcttctactggcggctga-3′
	r: 5′-agggttggcattgtggatt-3′
ACLY	f: 5′-accgaagaccaacatccaca-3′
	r: 5′-gcaacatcctaacgccctac-3′
ALDOA	f: 5′-cgagaacaccgaggagaacc-3′
	r: 5′-tcagcacacaacgccactt-3′
FASN	f: 5′-ccaaggacacagtcaccatct-3′
	r: 5′-ctgctccacgaactcaaaca-3′
GLS	f: 5′-gctgtgctccattgaagtgact -3′
	r: 5′- ttgggcagaaaccaccattag-3′
GLUT1	f: 5′-cttctatcccaggaggtggctat-3′
	r: 5′- aatggagcctgacccctagag-3′
GLUT3	f: 5′-gccgctgctactgggtttta-3′
	r: 5′-gggactttcagggcaaaatg-3′
HIF1A	f: 5′-ttccagttacgttccttcgatca -3′
	r: 5′-tttgaggacttgcgctttca -3′
HIF2A	f: 5′-gtgctcccacggcctgta-3′
	r: 5′-ttgtcacacctatggcatatcaca-3′
HK2	f: 5′-gccgggctgagattcttct-3′
	r: 5′-gcgacgtgtccggttgtc-3′
HPRT1	f: 5′-tgacactggcaaaacaatgca-3′
	r: 5′-ggtccttttcaccagcaagct-3′
LDHA	f: 5′-agcccgattccgttacct-3′
	r: 5′-gcaacattcattccactcca-3′
IDH1	f: 5′-tcagtggcggttctgtggta-3′
	r: 5′-cttggtgacttggtcgttggt-3′
MYC	f: 5′-cgtctccacacatcagcacaa-3′
	r: 5′-cactgtccaacttgaccctcttg-3′
MYCL	f: 5′-tccacacccgtgagaaatcct-3′
	r: 5′-ttccataccccattcccca-3′
MYCN	f: 5′-cgcaaaagccacctctcatta-3´
	r: 5′-tccagcagatgccacataagg-3´
PDK1	f: 5′-ccagcagagaacccaaaga
	r: 5′-tccgatgtcccaagtgtgt
SLC1A5	f: 5′-actgcctttgggacctcttc
	r: 5′-attctcctccacgcacttca
SLC16A3	f: 5′-ctctccagcccgtcctacc
	r: 5′-tcctaacactccacccacac
TBP	f: 5′-cacgaaccacggcactgatt-3′
	r:5′-ttttcttgctgccagtctggac-3′
UBC	f: 5′-atttgggtcgcggttcttg-3′
	r: 5′-tgccttgacattctcgatggt-3′
